# Development and evaluation of an efficient heterologous gene knock-in reporter system in *Lactococcus lactis*

**DOI:** 10.1186/s12934-017-0770-1

**Published:** 2017-09-18

**Authors:** Yifei Lu, Hongxiang Yan, Jiezhong Deng, Zhigang Huang, Xurui Jin, Yanlan Yu, Qiwen Hu, Fuquan Hu, Jing Wang

**Affiliations:** Department of Microbiology, Third Military Medical University, Chongqing, 400038 China

**Keywords:** *Lactococcus lactis*, NZ9000, Knocked-in heterologous gene, Knock-in reporter system, *lacZ*

## Abstract

**Background:**

*Lactococcus lactis* is a food grade probiotics and widely used to express heterologous proteins. Generally, target genes are knocked into the *L. lactis* genome through double-crossover recombination to express heterologous proteins stably. However, creating marker-less heterologous genes knocked-in clones is laborious. In this study, an efficient heterologous gene knock-in reporter system was developed in *L. lactis* NZ9000.

**Results:**

Our knock-in reporter system consists of a temperature-sensitive plasmid pJW and a recombinant *L. lactis* strain named NZB. The pJW contains homologous arms, and was constructed to knock-in heterologous genes at a fixed locus of NZ9000 genome. *lacZ* (*β*-galactosidase) gene was knocked into the chromosome of NZ9000 as a counter-selective marker through the plasmid pJW to generate NZB. The engineered NZB strain formed blue colonies on X-Gal plate. The desired double-crossover mutants formed white colonies distinctive from the predominantly blue colonies (parental and plasmid-integrated clones) when the embedded *lacZ* was replaced with the target heterologous genes carried by pJW in NZB.

**Conclusions:**

By using the system, the heterologous gene knocked-in clones are screened by colony phenotype change rather than by checking colonies individually. Our new knock-in reporter system provides an efficient method to create heterologous genes knocked-in clones.

**Electronic supplementary material:**

The online version of this article (doi:10.1186/s12934-017-0770-1) contains supplementary material, which is available to authorized users.

## Background

*Lactococcus lactis*, a food-grade Gram-positive lactic acid bacterium, is commonly used to manufacture fermented dairy products, such as (soft) cheese, buttermilk, and sour cream [1, 2]. Since 1980s, extensive research on *L. lactis* has revealed considerable information on the biological, genetic, and immunological characteristics of this species [3, 4]. *L. lactis* has been broadly used as an “efficient cell factory” for recombinant protein production [5] because of the following properties: (i) As a generally regarded as safe (GRAS) microorganism [1], *L. lactis* elicits weak immune responses against itself and does not colonize the gut of humans and animals [6]. Thus, *L. lactis* can be directly used in the digestive tract [7, 8]. (ii) *L. lactis* is genetically easy to manipulate, because of its completely sequenced genome [9–11] and many available genetic tools [3, 5, 12]. (iii) The downstream purification processes of secreted recombinant proteins are simple because *L. lactis* secretes only one major protein, namely, Usp45 [5]. Several kinds of heterologous proteins, such as enzymes [13–15], therapeutic proteins [16–18], growth factors [19–21], and antigens [3, 6, 12, 22], have been expressed in *L. lactis*. Therefore, *L. lactis* is a suitable host for heterologous gene expression and becomes the focus of food industry, biopharmaceuticals, and vaccine research.

Heterologous proteins can be expressed in *L. lactis* by encoding their genes harbored in vectors, such as pNZ8148 [3, 23], pMG36e [15, 24], pAMJ399 [19–21], and pLEB590 [25, 26]. However, this approach is limited by several disadvantages. (i) In these vectors, antibiotic-resistant genes, which are banned for use in humans, are commonly employed as selective markers. (ii) Food-grade selective markers, such as nisin resistance gene (*nsr*), have been applied in *L. lactis*. However, most of the food-grade selective markers cannot be used in *Escherichia coli*. Therefore, plasmids containing these food-grade selective markers can only be constructed in *L. lactis*, but the efficiency of constructing plasmids in *L. lactis* is much lower than that in *E. coli*. (iii) Plasmids in *L. lactis* are unstable in human and animal digestive tracts in the abundance of selective pressure. As an efficient alternative approach, the knock-in of target genes into *L. lactis* chromosome through double-crossover recombination is performed to stably express heterologous proteins without antibiotic-selective markers.

Temperature-sensitive (Ts) plasmids are usually utilized to integrate heterologous genes into the *L. lactis* genome. The entire process is accomplished in two steps [27]. First, a Ts plasmid harboring a target heterologous gene is transformed into *L. lactis*. Single-crossover recombinants are then obtained by culturing the transformants with antibiotics at a nonpermissive temperature. Second, plasmid-integrated clones are grown at a permissive temperature in an antibiotic-free medium. The integrated vector can be excised from the genome at a low frequency through a second recombination and consequently produce wild-type or heterologous gene knocked-in (HGK) strain without antibiotic resistance. To screen non-resistant clones, we individually examine the antibiotic resistance provided by integrated plasmids in colonies. HGK clones are subsequently checked through PCR. However, screening is laborious and time consuming, that is, this process requires several days to weeks. Therefore, a rapid screening method for HGK clones is desirable.

In this study, a heterologous gene knock-in reporter system was established for *L. lactis* NZ9000 (*β*-galactosidase negative strain) through visual selection. The proposed system comprised a Ts pJW plasmid and a recombinant *L. lactis* NZB strain. pJW contains homologous arms, and was constructed to knock-in heterologous genes at a fixed locus of NZ9000 genome. Afterward, the *lacZ* (*β*-*galactosidase*) gene was knocked-in the chromosome of NZ9000 by pJW. The resulting mutant strain, named NZB, formed blue colonies on X-Gal (5-bromo-4-chloro-3-indolyl-β-d-galactopyranoside) plate. To knock a target gene into *L. lactis* chromosome through the knock-in reporter system, the heterologous gene was firstly inserted in pJW. Then, the pJW vector harboring the target gene was transformed into NZB. When the heterologous gene was knocked into the NZB chromosome and replaced the *lacZ* gene by double-crossover recombination, the HGK clones formed white colonies on X-Gal plate and were distinguished from the other blue colonies. The HGK clones were then screened by selecting the white colonies from blue colonies rather than by checking colonies individually. By utilizing our knock-in reporter system, the HGK clones were produced simply and efficiently.

## Results

### Construction of the Ts plasmid pJW

**Fig. 1:**
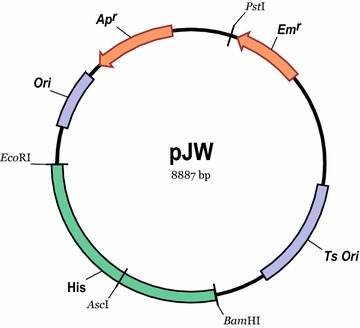
Map of the Ts plasmid pJW. His, fragment from *L. lactis* as homologous arms; *Ts Ori*, temperature-sensitive replicon from pCrePA2; *Ori*, replicon from pUC18; *Em* ^*r*^, erythromycin resistance gene; *Ap* ^*r*^, ampicillin resistance gene

pJW, a Ts vector, was constructed and used in subsequent experiments. This vector was based on pUC18 added with a Ts replication origin RepA(Ts) [27, 28], an erythromycin resistance gene from pCrePA2 [29, 30], and a unified homologous sequence with an *Asc*I restriction site at the center (Fig. 1). The homologous sequence was an internal 2.5 kb-fragment of the histidine operon of *L. lactis* NZ9000 (*His* locus) [31] and used for recombination. The heterologous genes were inserted into the *Asc*I site by restriction digestion and ligation, and the fragment was divided into two parts (Hisa and Hisb). These two parts then served as homologous arms for recombination in *L. lactis* [32]. If the *Asc*I restriction site is present in the gene of interest, the gene can be inserted into the *Asc*I site by a seamless cloning strategy. pJW contains ampicillin- and erythromycin-resistant genes.

### Construction of NZB strain

We first integrated a *lacZ* gene with a promoter into the *His* locus of the chromosome of NZ9000 because *β*-galactosidase gene is absent in the NZ9000 chromosome. The PZLT fragment containing a nisin promoter *P*_*nisZ*_ from *L. lactis* N8 [33], *lacZ* from *Lactobacillus acidophilus* [34], and a terminator from the pMG36e plasmid [15, 35] was cloned into pJW to create pJW-PZLT (Additional file 1: Figure S2). *P*_*nisZ*_, which can be induced in the presence of nisin in NZ9000, was artificially synthesized on the basis of the reference sequence [33].

**Fig. 2:**
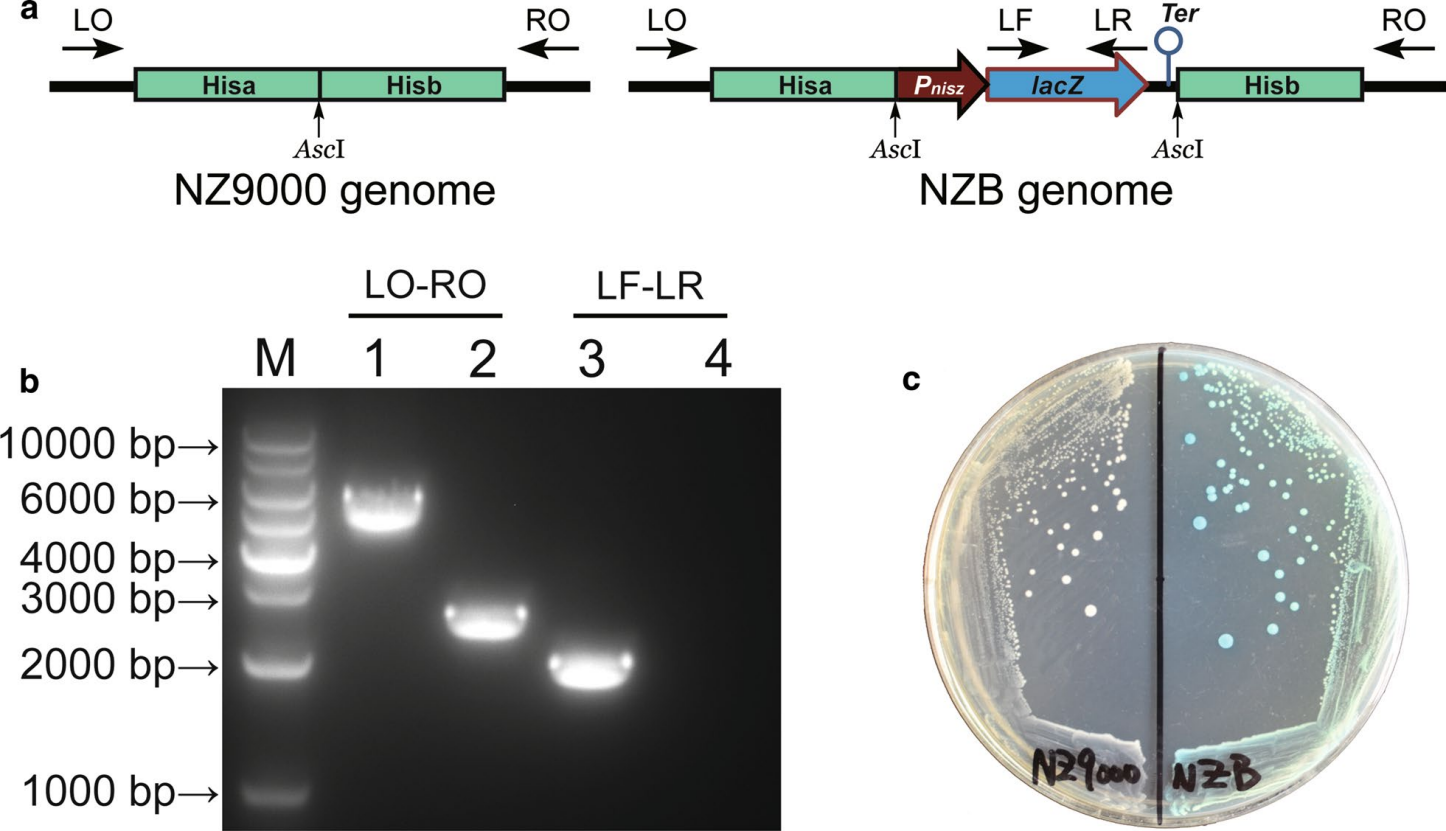
Confirmation analysis of the NZB strain. **a** Schematic of genomes of NZ9000 and NZB. LO, RO, LF, LR, primers used in multiple-PCR analysis. **b** Multiple-PCR analysis of the NZB and NZ9000 strain. The primer combinations used in PCR are presented on the lanes. Genomic DNA from the following strains were used as templates: NZB (lanes 1 and 3) and NZ9000 (lanes 2 and 4). The 1 kb DNA ladder marker is shown on the left (M). The theoretical size (bp) of each PCR products generated with the primer combinations is shown in Additional file 1: Table S1. **c** NZ9000 (left) and NZB (right) colonies on M17GS-XN

The PZLT expression cassette was knocked into NZ9000 chromosome by pJW-PZLT plasmid. The blue colonies were screened separately by erythromycin-resistance test. Erythromycin-sensitive colonies represent the strains with plasmid region excised by double-crossover event and eliminated by culturing at 37 °C. The erythromycin-sensitive blue colonies were then checked by multiple-PCR analysis (Fig. 2a), and all PCR products (Fig. 2b) corresponded to the theoretical sizes (Additional file 1: Table S1). Subsequent sequencing of PCR products amplified with primers pairs specific to the genomic regions flanked by the homologous arms (LO-RO) confirmed that the PZLT fragment was knocked into the *L. lactis* genome. The engineered strain was named NZB which formed blue colonies on the M17GS-XN plate (Fig. 2c).

### Evaluating the efficiency of heterologous gene knock-in reporter system in *L. lactis*

The “efficient heterologous gene knock-in reporter system in *L. lactis*” is composed of the pJW plasmid and the NZB strain. To integrate a heterologous gene into the NZB chromosome, we used the built-in *lacZ* gene as a target counter-selection marker. The NZB strain loses its ability to produce *β*-galactosidase and forms white colonies that are easily distinguished from the blue parental colonies on an X-Gal plate when the built-in *lacZ* gene is replaced by the gene of interest via double-crossover recombination.

**Fig. 3:**
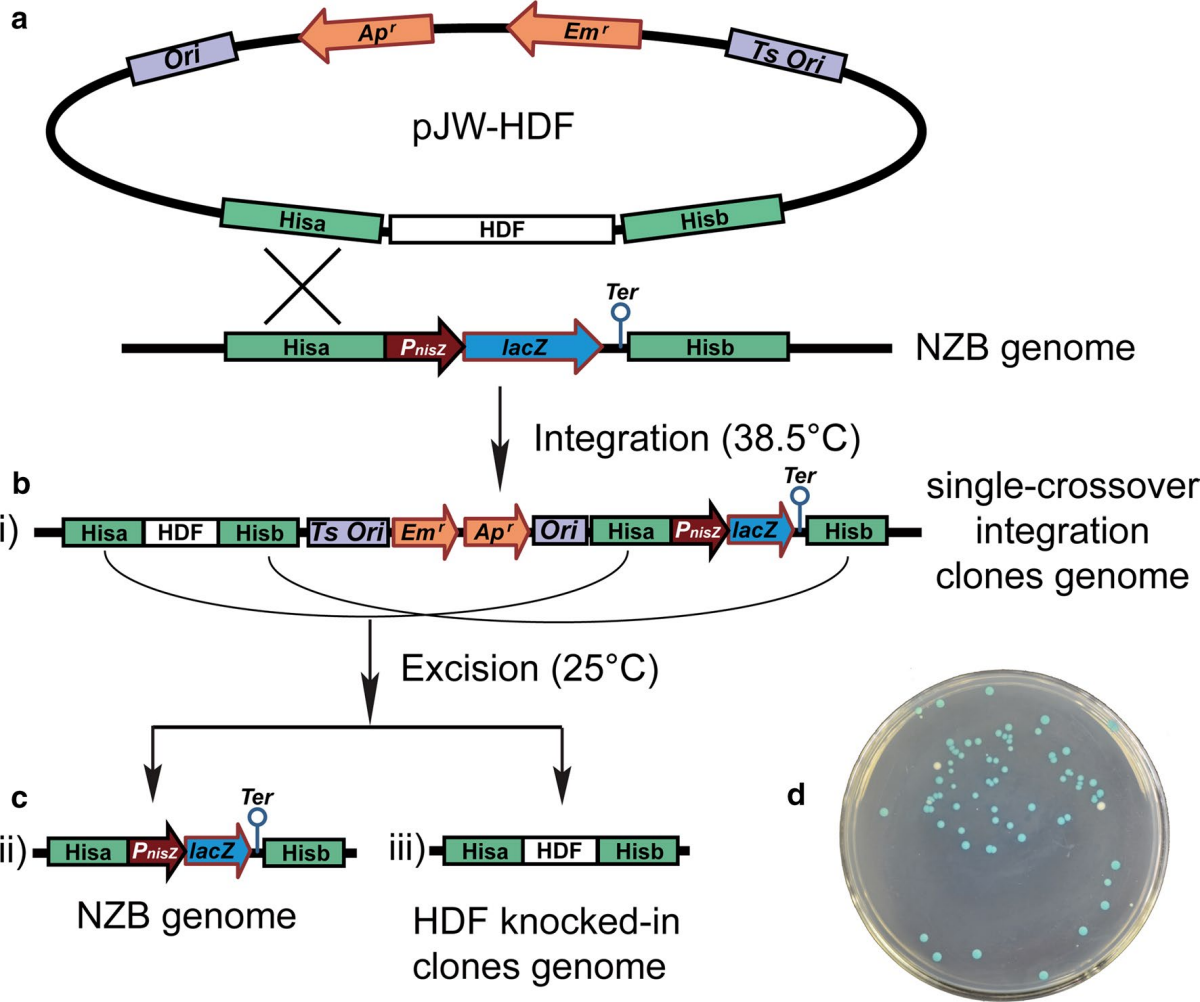
Schematic of the knock-in reporter system. **a** A pJW-HDF vector (derivative of pJW) was introduced into NZB at 30 °C. **b** The single-crossover recombination colonies were selected at 38.5 °C with erythromycin. The single-crossover recombination event occurred in either Hisa region or Hisb region (not shown in schematic). **c** At 25 °C without antibiotic, the plasmid region in the genome was excised through a second recombination. At the statuses i and ii, the colonies were blue on M17GS-XN plate. At status iii, the colonies were white on M17GS-XN plate. **d** Representative white colonies (HGK clones) from the blue colonies (plasmid-integrated clones and NZB clones). HDF, heterologous DNA fragment

To evaluate the feasibility and efficiency of the knock-in reporter system, five heterologous DNA fragments (HDFs) varied from ~1.3 to ~14.6 kb were integrated into the chromosome of *L. lactis* NZB by using the knock-in reporter system. The five HDFs of interest were inserted in the *Asc*I site of pJW by seamless cloning, creating pJW-1.3, pJW-2.2, pJW-3.8, pJW-7.3, and pJW-14.6, respectively. After the HDF-containing plasmids were integrated into NZBs chromosome (Fig. 3b), the plasmid-integrated clones were then grown in the M17GS medium without antibiotic overnight at 25 °C (Fig. 3c), and plated on M17GS-XN at 37 °C until most colonies turned blue. Colonies were formed on the plates from the following three cases. The second recombination did not occur produced plasmid-integrated clones (i); the plasmids were excised via a second allelic exchange and resulted in parental clones NZB (ii), or HGK clones (iii) (Fig. 3b, c). For (i) and (ii), the clones contained the *lacZ* gene, and the colonies were blue on M17GS-XN agar. Only in (iii), were the *lacZ* gene replaced by HDFs, and the resultant colonies (HGK clones) were white on M17GS-XN agar (Fig. 3d).

**Fig. 4:**
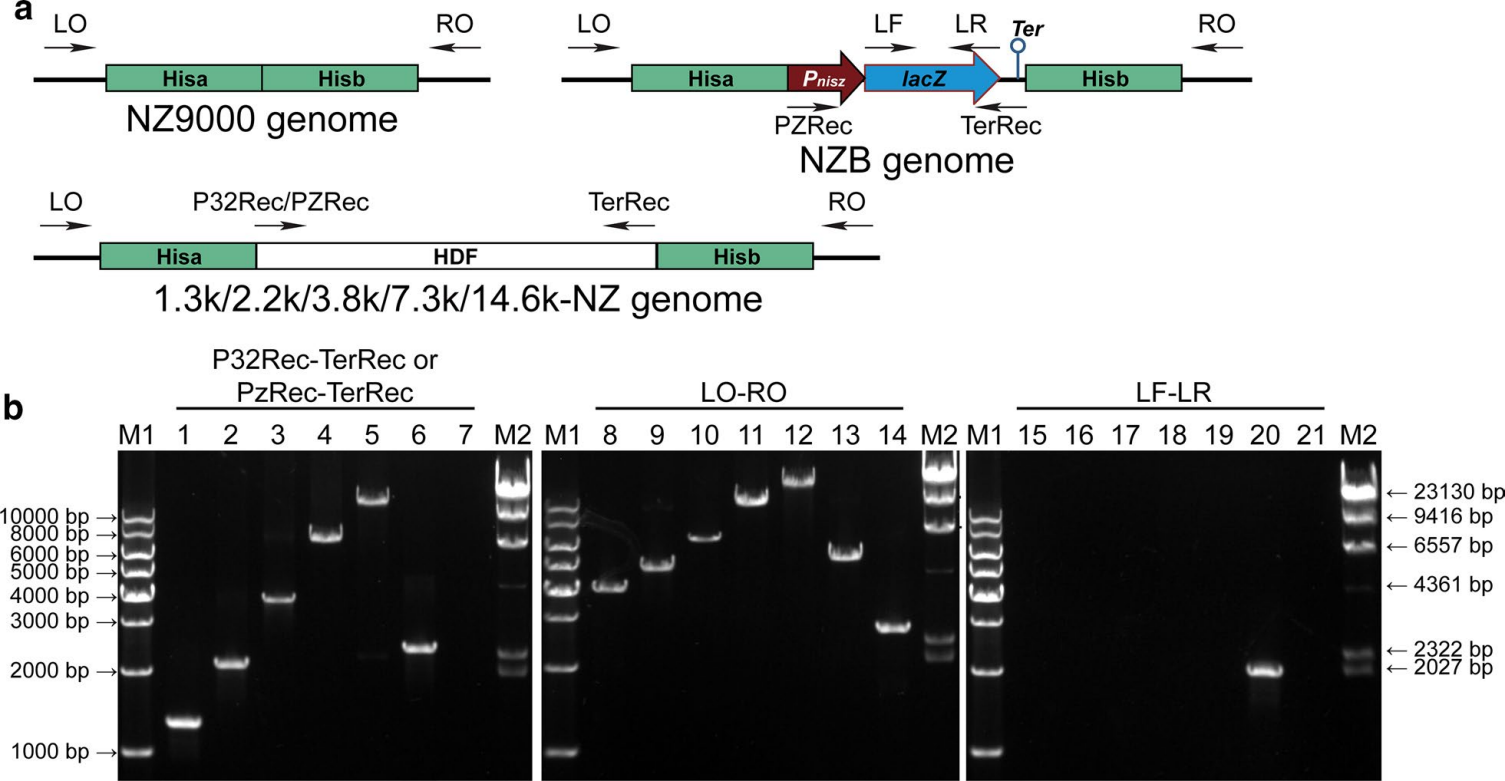
Confirmation analysis of the five HDFs knocked-in strains constructed through the knock-in reporter system. **a** Schematic of genomes of the NZ9000, NZB, and the five HDF knocked-in strains. LO, RO, LF, LR, P32Rec, PZRec, and TerRec as primers used in Multiple-PCR analysis. **b** Confirmation analysis of the HDFs knocked-in strains by multiple-PCR analysis. The primer combinations used in PCR are presented on the lanes. Genomic DNA from the following strains were used as templates: 1.3k-NZ (lanes 1, 8, and 15), 2.2k-NZ (lanes 2, 9, and 16), 3.8k-NZ (lanes 3, 10, and 17), 7.3k-NZ (lanes 4, 11, and 18), 14.6k-NZ (lanes 5, 12, and 19), NZB (lanes 6, 13, and 20) and NZ9000 (lanes 7, 14, and 21). The 1 kb DNA ladder marker is shown on the left (M1), and the λ/*Hin*dIII marker is shown on the right (M2). The theoretical size (bp) of each PCR products generated with the primer combinations is shown in Additional file 1: Table S1. HDF, heterologous DNA fragment; LO, RO, LF, LR, P32Rec, PZRec, and TerRec as the primers used in Multiple-PCR analysis

These white colonies were then verified by multiple assays. First, all the white colonies were sensitive to erythromycin; hence, the erythromycin-resistant integrated plasmids were excised and lost. Second, the white colonies were verified by multiple-PCR analysis (Fig. 4a), and all PCR products (Fig. 4b) corresponded to the theoretical sizes (Additional file 1: Table S1). Subsequent sequencing of the PCR products amplified with primers pairs specific to the genomic regions flanked by the homologous arms (LO-RO) confirmed that the HDFs were knocked into the NZB genomes and replaced the *lacZ* gene. The HDFs were named 1.3k-NZ, 2.2k-NZ, 3.8k-NZ, 7.3k-NZ, and 14.6k-NZ. We counted the white colony occurrence rate and accuracy rate. Although the white colonies occurrence rate was extremely low (10^−3^ to 10^−5^), the accuracy rate was 100% as expected (Table 1).

**Table 1:** White colony occurrence and accuracy rate in the evaluation of the knock-in reporter system

Name	HDF length (bp)	Total colonies	White colonies	White colony occurrence rate (‰)	Accuracy rate (%)
1.3k-NZ	1317	6765	58	8.873	100.00
2.2k-NZ	2163	7508	44	5.860	100.00
3.8k-NZ	3856	15,239	21	1.378	100.00
7.3k-NZ	7277	14,599	12	0.8220	100.00
14.6k-NZ	14,620	16,349	3	0.1835	100.00

In conclusion, our knock-in reporter system can effectively knock-in the HDFs ranging from 1.3 to 14.6 kb into the *L. lactis* chromosome. Furthermore, the white colonies were HGK colonies, and the accuracy rate was 100%.

## Discussion

In this work, we developed an efficient heterologous gene knock-in reporter system in *L. lactis*. The knock-in reporter system contains a Ts plasmid pJW and a NZB strain (a derivative of NZ9000). By using the knock-in reporter system, the HDF knocked-in NZB clones can be selected directly by colony phenotype (color) change (blue to white). Our assay largely reduced the required labor and time, and thus improved the efficiency of knock-in assay for *L. lactis*.

In the original assay, the knocked-in strains were screened by examining the loss of antibiotic resistance, hundreds and thousands of colonies must be examined individually to obtain antibiotic-sensitive clones. Some of these colonies were HDF knocked-in clones, and others were parental clones. In our assay, screening was simplified through visual observation of phenotypic colony changes, and the screening time was reduced from several weeks to several days.

The parental strain used in this study was NZ9000 (*L. lactis* subsp. *cremoris*) [11], a derivative of MG1363, and is the most commonly used host of the NIsin Controlled gene Expression (NICE) system. The NICE system is a highly successful and widely used tool for regulating of gene expression in *L. lactis* [11, 23, 36, 37]. When a gene of interest is placed at the downstream of the inducible promoter *P*_*nisA*_, *P*_*nisF*_, or *P*_*nisZ*_ in NZ9000, industrial-scale gene expression can be induced by adding nisin (a food-grade antimicrobial peptide produced by some strains of *L. lactis*) to the culture medium [36–38]. Besides nisin inducible promoters, other promoters, like constitutive promoters *P*_*32*_ [39] and *P*_*45*_ [33], and pH inducible promoter *P*_*170*_ [40], can also work in NZ9000. Therefore, NZ9000 was chosen to develop the host strain of the knock-in reporter system instead of other *L. lactis* strains.

Two kinds of plasmids namely, non-replicative and Ts plasmids, are employed in gene knockout and knock-in. Non-replicative plasmids can be maintained by the target host only by specific recombination with the host genome, and the resulted clones are selected using antibiotics. Obtaining plasmid-integrated clones is difficult in certain hosts, such as *L. lactis*, because of low transformation efficiency. Owing to its large size, Ts plasmids are difficult to use in transforming target hosts. However, this kind of plasmids can be replicated in the target host at a permissive temperature. Transformants are easily achieved, and only one transformant is needed for subsequent operations. Therefore, we chose Ts plasmid as a vehicle of the gene of interest in our knock-in reporter system.

In the present study, the *His* locus, corresponding to a nonessential gene involved in histidine biosynthesis in *L. lactis* [31], was employed as the integration site, which was also described in a previous study by Simões-Barbosa et al. [32]. Their results demonstrated that the disruption of the *His* locus did not significantly affect growth of the heterologous gene integrated strain [32], which was confirmed by our results. Besides the *His* locus, *thyA*, a thymidylate synthase-encoding gene, was also used as integration site for the construction of delivery system in vivo [17, 41]. Replacement of *thyA* by heterologous genes creates *thyA*-deficient strains, which are self-limited and die rapidly in the absence of thymidine or thymine. Thus, the *thyA*-deficient bacteria can survive in vivo, but cannot accumulate in the environment [17].

This system can be only applied in *β*-galactosidase negative and *nisRK*-positive strains, such as NZ9000, because the nisin inducible promoter *P*_*nisZ*_ can only work in *nisRK*-positive strains. However, we believe that, the strategy can also be applied in other hosts. For *β*-galactosidase and *nisRK* genes negative strains, the nisin inducible promoter *P*_*nisZ*_ in *lacZ* expression cassette should be replaced by constitutive promoters or other kind of inducible promoters (depend on host strains) [3]. For *β*-galactosidase positive strains, the *β*-galactosidase gene should be used as counter-selective marker.

## Conclusion

In conclusion, the “efficient heterologous gene knock-in reporter system in *L. lactis*” is a convenient and practical tool to knock heterologous genes into *L. lactis* NZB strain (a derivative of NZ9000) efficiently, and enhances downstream research works.

## Methods

### Bacteria, plasmids, and culture conditions

All the bacteria strains and plasmids used in this study are listed in Table 2 and Additional file 1: Table S2. *E. coli* strain DH5α was used as cloning host and cultured in Luria–Bertani (LB, Oxoid, UK) medium at 37 °C. *L. lactis* was cultured in M17GS (M17 [Oxoid, UK] supplemented with 0.5% [wt/vol.] glucose, 0.5% [wt/vol.] lactose, 0.55% [wt/vol.] sucrose) medium at 30 °C. Ampicillin (100 μg/mL, Tiangen, China) was used for *E. coli*. Erythromycin (20 μg/mL, BBI, Canada) was used for *L. lactis*. X-Gal (BBI, Canada) and nisin (Sigma-Aldrich, USA) were utilized at 40 and 0.1 μg/mL, respectively, for screening in agar.

**Table 2:** Bacterial strains and plasmids used in this study

Strain	Characteristics	Source
*E. coli* DH5α	Cloning host for maintaining recombinant plasmids	Lab collection
*L. lactis* NZ9000	Derivative of MG1363; *pepN::nisRK*	[37], Lab collection
*L. lactis* NZB	Derivative of NZ9000; *P*_*nisZ*_ *::lacZ::terminator*	This study
Plasmids		
pUC18	Cloning vector; *Ap* ^*r*^	Lab collection
pUC-H	Derivative of pUC18; containing His fragment from *L. lactis*	This study
pCrePA2	An improved version of the pCrePA [29] plasmid containing Ts replicon; *Ap* ^*r*^, *Em* ^*r*^	Kindly gifted by Stephen H. Leppla
pJW	*L. lactis* integration vector; derivative of pUC-H; containing Ts replicon from pCrePA2; *Ap* ^*r*^, *Em* ^*r*^	This study
pMG36e	Wide-host-range vector; *Em* ^*r*^	[15], Lab collection
pQE31-*LacZ*	Derivative of the expression vector pQE31; containing *lacZ* from *Lactobacillus acidophilus*; *Ap* ^*r*^	[34], Lab collection
pMG-PZL	derivative of pMG36e; containing *P*_*nisZ*_ *::lacZ*; template of *P*_*nisZ*_ *::lacZ::terminator*; *Em* ^*r*^	This study
pJW-PZLT	*L. lactis* integration vector; derivative of pJW; containing *P*_*nisZ*_ *::lacZ::terminator*; *Ap* ^*r*^, *Em* ^*r*^	This study

*Ap* ^*r*^ ampicillin resistant, *Em* ^*r*^ erythromycin resistant, *Ts* temperature-sensitive

### DNA manipulations and sequencing

The construction of plasmid pJW was performed in *E. coli*. The PCR primers used in this study are listed in Table 3. PCR products were purified with the Wizard SV Gel and PCR Clean-Up System (Promega, USA). Plasmids were isolated with the Plasmid Mini Kit (Omega Bio-tek, USA). The restriction enzymes were FastDigest Restriction Enzymes (Thermo Scientific, USA). Genomes were isolated with the TIANamp Bacteria DNA Kit (Tiangen, China). PCR products and plasmids were sequenced by the Beijing Genomics Institute (China). Seamless cloning was performed with the NovoRec PCR One-Step Directed Cloning kit (Novoprotein, China). Molecular manipulation [42], preparation of competent cells [43], and electrotransformation [43] was performed as described previously.

**Table 3:** Primers used in this study

Primers	Sequence (5′–3′)	Restriction sites
HisF	AAA**GAATTC**TAAAGTAATTTTCATCAATTTTTTCTAAGC	*Eco*RI
HisR	GTTTGGGAGTCGCCTTTGGCTC	–
TsF	AAA**GGATCC**TGATCGTTAAATTTATACTGCAAT	*Bam*HI
TsR	AAA**CTGCAG**TACCTAATAATTTATCTACATTCCC	*Pst*I
PZF	AAA**GAATTC**AGTCTTATAACTATACTGACAATAG	*Eco*RI
PZR	TGATAATTGTGTCATTTTGAGTGCCTCCTTATAAT	–
LF	AAGGAGGCACTCAAAATGACACAATTATCACGTTT	–
LR	AAA**TCTAGA**CTAATTTCTCAATACTTGAACAT	*Xba*I
P32Rec	AAGTCGCGT**GGCGCGCC**GGTCCTCGGGATATGATA	*Asc*I
PZRec	AAGTCGCGT**GGCGCGCC**AGTCTTATAACTATACTGACAATAG	*Asc*I
TerRec	GAAATGATA**GGCGCGCC**ATAAGCAAAAGGCAGCTGAT	*Asc*I
LO	GCTCCATGAATTTCCTAATTGATGC	–
RO	GATGAAGCTTATATTGACTTTGGCG	–

Restriction enzyme cut sites are in bold

### Construction of Ts plasmid pJW

The His fragment (2472 bp), an internal sequence of the histidine operon, was amplified from the genome of NZ9000 by PCR using PrimerStar DNA polymerase (high-fidelity DNA polymerase, Takara, China) with primers HisF and HisR from NZ9000 genome. The His fragment was then digested with *Eco*RI, and inserted in the *Sma*I/*Eco*RI sites of pUC18. The resulting vector was named pUC-H. The “*Ts*-*replicon::Em* ^*r*^” fragment amplified from pCrePA2 vector with primers TsF and TsR was digested and inserted in the *Bam*HI/*Pst*I sites of pUC-H. The constructed vector was named pJW (Additional file 1: Figure S1).

### Construction of NZB strain

The nisin promoter *P*_*nisZ*_ [33] was artificially synthesized as template, and was amplified with the primers PZF and PZR. The *lacZ* gene (from *L. acidophilus*) was amplified from pQE31-*LacZ* [34] with the primers LF and LR. *P*_*nisZ*_ and *lacZ* gene were combined by overlap PCR. The PCR product was digested by *Eco*RI/*Xba*I and cloned into the same sites of pMG36e [15] to yield the plasmid pMG-PZL. The PZLT fragment (*P*_*nisZ*_ *::lacZ::terminator*) was then amplified from pMG-PZL with primers PZRec and TerRec and inserted into the *Asc*I site of pJW by seamless cloning (Additional file 1: Figure S2).

The vector pJW-PZLT was introduced into *L. lactis* NZ9000 by electroporation, and the transformants were selected at 30 °C on M17GS-NX (M17GS agar containing X-Gal and nisin) medium containing erythromycin (Additional file 1: Figure S3A). One blue colony was streaked onto the same medium and incubated at 30 °C. In the next step, a blue colony was inoculated in M17GS broth with erythromycin and incubated at 30 °C for 8 h and then diluted 1000-fold in the same medium and grown at nonpermissive temperature (38.5 °C) overnight to select the chromosomal-plasmid-integrated strain (Additional file 1: Figure S3B). Next, the cultures were then diluted 1:10^6^ in M17GS medium without antibiotic and grown overnight at permissive temperature (25 °C) to stimulate a second recombination event [27], and the plasmid was excised from chromosome (Additional file 1: Figure S3C). Dilutions of the overnight cultures were plated on M17GS-XN and incubated at 37 °C to eliminate the excised Ts plasmid. Single blue colonies were screened by replica plating on M17GS-XN plates versus M17GS-XN plates containing erythromycin. The erythromycin-sensitive blue colonies represented the strains with a plasmid region excised in a double-crossover event and the PZLT fragment inserted in the NZ9000 genome. The resultant stain was named NZB (Additional file 1: Figure S3).

### Evaluating the efficient heterologous gene knock-in reporter system in *L. lactis*

To evaluate the knock-in reporter system, we knocked five different lengths (1.3–14.6 kb) of DNA fragments into the *L. lactis* NZB genome as heterologous genes. The five fragments were amplified from the vectors of our laboratory’s collection with the primer pair P32Rec–TerRec or PZRec–TerRec and cloned into the vector pJW through its *Asc*I site by seamless cloning. The constructed vectors were named pJW-1.3, pJW-2.2, pJW-3.8, pJW-7.3, and pJW-14.6 (Additional file 1: Table S2.). The five vectors were transformed into NZB, and the transformants were selected at 30 °C on M17GS-NX medium containing erythromycin (Fig. 3a). The plasmids harboring NZB were inoculated in M17GS broth with erythromycin and incubated at 30 °C for 8 h, and were then diluted at 1:10^3^ in the same medium and incubated at 38.5 °C to select plasmid-integrated clones (Fig. 3b). The overnight cultures were then diluted at 1:10^6^ in M17GS medium without antibiotic and grown overnight at 25 °C (Fig. 3c). The overnight cultures were diluted and plated on M17GS-XN at 37 °C until most colonies turned blue. The white colonies were picked for further identification.

## Additional file

**Additional file 1.** Additional tables and figures.
