# Supplementary material for: Development and evaluation of an efficient heterologous gene knock-in reporter system in Lactococcus lactis
Source: Microb Cell Fact. 2017 Sep 18;16:154. doi: 10.1186/s12934-017-0770-1 (PMC5604289; doi:10.1186/s12934-017-0770-1)
Supplement: Supplementary file 1 — Additional file 1. Additional tables and figures. [file 12934_2017_770_MOESM1_ESM.doc]

**Development and Evaluation of an Efficient Heterologous Gene Knock-in Reporter System in *Lactococcus lactis***

Yifei Lu, Hongxiang Yan, Jiezhong Deng, Zhigang Huang, Xurui Jin, Yanlan Yu, Qiwen Hu, Fuquan Hu, Jing Wang

Department of Microbiology, Third Military Medical University, Chongqing 400038, China

Table S1 Theoretical size (bp) of each PCR product generated with the primer combinations used in the multiple-PCR analysis

| Templates | Primer combinations | | | |
| --- | --- | --- | --- | --- |
|  | LO/RO | P32Rec/TerRec | PZRec/TerRec | LF/LR |
| NZ9000 | 2660 | - | - | - |
| NZB | 5124 | - | 2464 | 2004 |
| 1.3k-NZ | 3977 | 1317 | - | - |
| 2.2k-NZ | 4823 | 2163 | - | - |
| 3.8k-NZ | 6516 | 3856 | - | - |
| 7.3k-NZ | 9937 | 7277 | - | - |
| 14.6k-NZ | 14620 | - | 17280 | - |

#### **Table S2 Bacterial strains and plasmids used in this study.**

| Strain | Characteristics | Source |
| --- | --- | --- |
| *L. lactis* 1.3k-NZ | Derivative of NZB; containing 1317 bp heterologous DNA fragment (*P_32_::P_nisZ_::mcherry::Ter*) instead of *P_nisZ_::lacZ::terminator* | This study |
| *L. lactis* 2.2k-NZ | Derivative of NZB; containing 2163 bp heterologous DNA fragment (*P_32_::P_nisZ_::mcherry::nsr::Ter*) instead of *P_nisZ_::lacZ::terminator* | This study |
| *L. lactis* 3.8k-NZ | Derivative of NZB; containing 3856 bp heterologous DNA fragment (*P_32_::P_nisZ_::sp310m2::sct::pam::nsr::Ter*) instead of *P_nisZ_::lacZ::terminator* | This study |
| *L. lactis* 7.3k-NZ | Derivative of NZB; containing 7277 bp heterologous DNA fragment (*P_32_::P_nisZ_::luxCDABE::nsr::Ter*) instead of *P_nisZ_::lacZ::terminator* | This study |
| *L. lactis* 14.6k-NZ | Derivative of NZB; containing 14620 bp heterologous DNA fragment (*P_nisZ_::viaB::nsr::Ter*) instead of *P_nisZ_::lacZ::terminator* | This study |
| Plasmids | | |
| pJW-1.3 | *L. lactis* Ts vector for heterologous DNA fragment knock-in; derivative of pJW; containing 1317 bp heterologous DNA fragment (*P_32_::P_nisZ_::mcherry::Ter*); *Ap^r^*, *Em^r^* | This study |
| pJW-2.2 | *L. lactis* Ts vector for heterologous DNA fragment knock-in; derivative of pJW; containing 2163 bp heterologous DNA fragment (*P_32_::P_nisZ_::mcherry::nsr::Ter*); *Ap^r^*, *Em^r^* | This study |
| pJW-3.8 | *L. lactis* Ts vector for heterologous DNA fragment knock-in; derivative of pJW; containing 3856 bp heterologous DNA fragment (*P_32_::P_nisZ_::sp310m2::sct::pam::nsr::Ter*); *Ap^r^*, *Em^r^* | This study |
| pJW-7.3 | *L. lactis* Ts vector for heterologous DNA fragment knock-in; derivative of pJW; containing 7277 bp heterologous DNA fragment (*P_32_::P_nisZ_::luxCDABE::nsr::Ter*); *Ap^r^*, *Em^r^* | This study |
| pJW-14.6 | *L. lactis* Ts vector for heterologous DNA fragment knock-in; derivative of pJW; containing 14620 bp heterologous DNA fragment (*P_nisZ_::viaB::nsr::Ter*); *Ap^r^*, *Em^r^* | This study |

Abbreviations: *Ap^r^*, ampicillin resistant; *Em^r^*, erythromycin resistant; Ts, temperature-sensitive. *P_32_,* promoter (accession no. M24764.1); *P_nisZ_*, promoter (accession no. Y13384.1); *Ter*, terminator from plasmid pMG36e (); *mcherry*, fluorescent protein (accession no. KF790910.1); *nsr*, nisin resistance gene (accession no. U25181.1); *sp310m2*, signal peptide from NZ9000 (); *sct*, salmon calcitonin (accession no. XP_010888827.1); *pam*, peptidylglycine alpha-amidating monooxygenase from *Homo sapiens* (accession no. XM_017009510.1); *luxCDABE*, *lux* operon from *Vibrio harveyi* (accession no. JF420888.1); *viaB*, Vi polysaccharide synthesis operon from *Salmonella enterica* (AE014613.1).

Fig. S1


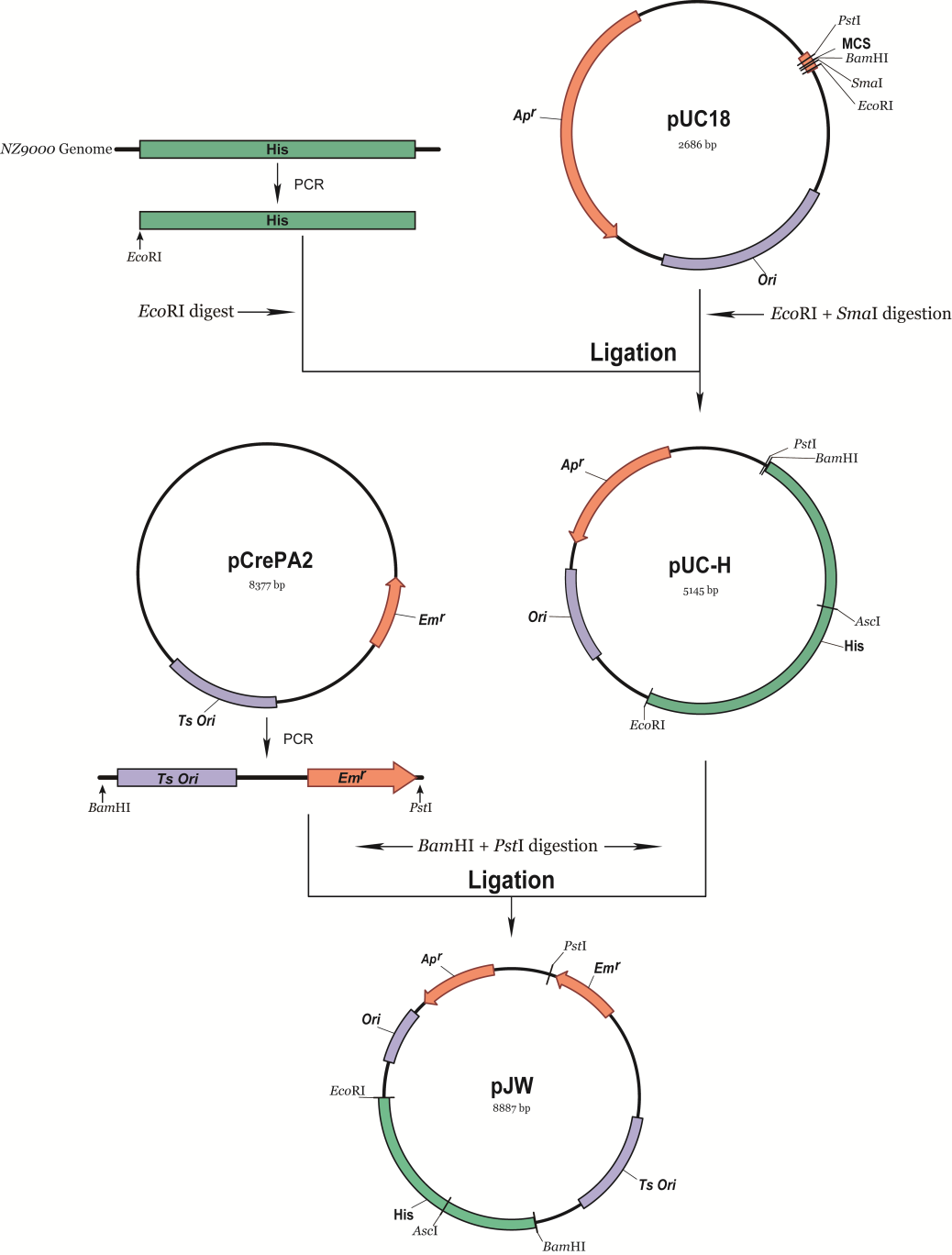


#### Fig. S1 Construction of pJW. Abbreviations: His, internal sequence of histidine operon from *L. lactis*, integration site of heterologous genes; *Ts Ori*, temperature-sensitive replicon from pCrePA2; *Ori*, replicon from pUC18; MCS, multiple clone site; *Em^r^*, erythromycin resistance gene; *Ap^r^*, ampicillin resistance gene.

Fig. S2


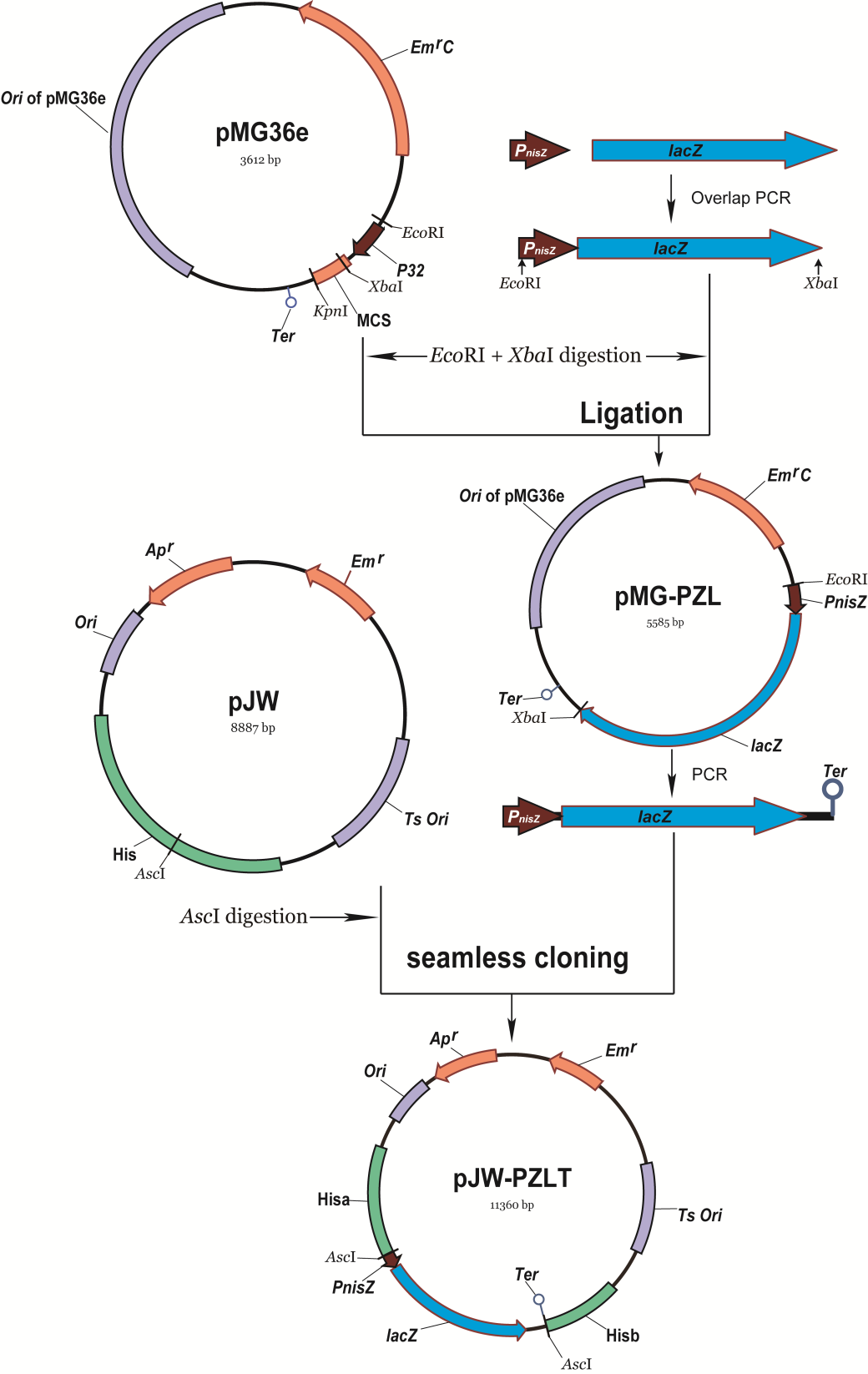


#### Fig. S2 Construction of pJW-PZLT. Abbreviations: His, internal sequence of histidine operon from *L. lactis*, knock-in site of heterologous gene; Hisa and Hisb, parts of His, homologous arms for homologous gene; *Ts Ori*, temperature-sensitive replicon from pCrePA2; *Ori*, replicon from pUC18; *Ori* of pMG36e, replicon of pMG36e; *P32*, *P_nisZ_*, promoter; *lacZ*, *β*-galactosidase from *Lactobacillus acidophilus*; MCS, multiple clone site; *Ter*, terminator from pMG36e; *Em^r^*, erythromycin resistance gene from pCrePA2; *Em^r^C*, erythromycin resistance gene of pMG36e; *Ap^r^*, ampicillin resistance gene.

Fig. S3


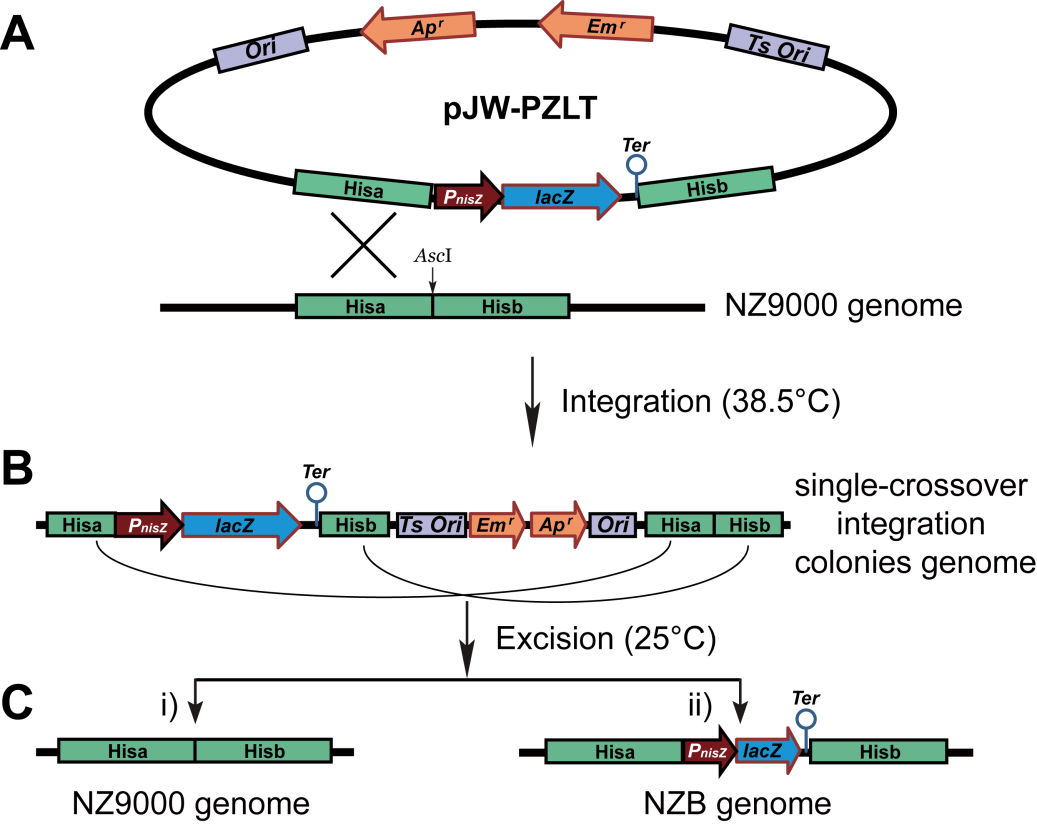


#### Fig. S3. Schematic of the construction of NZB. A). Plasmid pJW-PZLT was introduced into NZB at 30 °C. B). Plasmid integration into the genome by single-crossover recombination was selected at 38.5 °C with erythromycin. The single-crossover recombination event occurred in either Hisa region or Hisb region (not shown in the schematic). C). At 25 °C without antibiotic, the plasmid region in genome was excise by double-crossover recombination. Excision through region i). restored the parental genome, whereas excision through region ii). led to the NZB genome. Abbreviations: Hisa, Hisb, parts of His; *Ts Ori*, temperature-sensitive replicon from pCrePA2; *Ori*, replicon from pUC18; *P_nisZ_*, promoter *P_nisZ_*; *lacZ*, *β*-galactosidase from *Lactobacillus acidophilus*; *Ter*, terminator from pMG36e; *Em^r^*, erythromycin resistance gene; *Ap^r^*, ampicillin resistance gene.

#### **References**

1. van de Guchte M, van der Vossen JM, Kok J, Venema G. Construction of a lactococcal expression vector: expression of hen egg white lysozyme in *Lactococcus lactis* subsp. *lactis*. Appl Environ Microbiol. 1989; 55**:**224-8.

2. Ravn P, Arnau J, Madsen SM, Vrang A, Israelsen H. Optimization of signal peptide SP310 for heterologous protein production in *Lactococcus lactis*. Microbiology. 2003; 149**:**2193-201.
